# Staged Implant Rehabilitation Following Endodontic Treatment Failure of a Maxillary First Molar: A Case Report

**DOI:** 10.7759/cureus.112964

**Published:** 2026-07-19

**Authors:** Ninoska J Barrios Barrios, Lisandra Flores Machado, Fabiana Lora, Maray Folgoso, Awdrey Y Fernandez Saba

**Affiliations:** 1 Dentistry, Happy Smile, Miami, USA; 2 Dentistry, Corsar Dental, Hialeah, USA; 3 Dentistry, Family Dental Cosmetic Dentistry, Scarsdale, USA; 4 Dentistry, Real Smile, Miami, USA; 5 Dentistry, General Practice Office, Hallandale Beach, USA

**Keywords:** bone grafting, dental implant, endodontic failure, implant-supported crown, maxillary first molar, osseointegration, radiographic evaluation, root canal failure, scan body, socket preservation

## Abstract

Dental implants represent a reliable treatment option for replacing teeth with unfavorable prognoses due to failed endodontic treatment. Maintaining alveolar ridge dimensions after extraction is essential to achieve optimal implant positioning and long-term restorative success. This case report describes the radiographic progression of treatment in a 46-year-old male patient with a failed endodontically treated maxillary left first molar (tooth #14).

Radiographic evaluation demonstrated findings consistent with endodontic treatment failure, and extraction was recommended due to the tooth’s unfavorable long-term prognosis. Following atraumatic extraction, socket preservation was performed using a bone grafting procedure to maintain alveolar ridge dimensions. After approximately three months of healing, a dental implant was placed in the grafted site. An additional three-month healing period was allowed to facilitate osseointegration. A scan body was subsequently utilized to obtain a digital implant impression for the fabrication of the definitive restoration. Three weeks later, an implant-supported crown was delivered.

Serial radiographs documented the treatment sequence from endodontic failure through extraction, socket preservation, implant placement, digital impression acquisition, and delivery of the definitive implant-supported restoration. This report highlights the radiographic progression of staged implant rehabilitation following extraction of a failed endodontically treated maxillary left first molar. The educational value of this report lies in demonstrating how socket preservation can facilitate predictable delayed implant placement and successful restorative rehabilitation through a systematic, staged treatment approach.

## Introduction

Endodontic treatment is a highly predictable and effective procedure for preserving natural dentition; however, failures may occur because of persistent apical pathology, inadequate canal debridement, coronal leakage, missed root canal anatomy, or other biological and technical factors [1-3]. Persistent bacterial infection within the root canal system has been recognized as one of the principal causes of post-treatment apical periodontitis and long-term endodontic treatment failure, even when appropriate treatment protocols have been followed [4,5]. Although many failed cases can be managed through nonsurgical retreatment or endodontic microsurgery, some teeth ultimately require extraction because of persistent disease and an unfavorable long-term prognosis [6,7].

Following tooth extraction, preservation of alveolar ridge dimensions is an important consideration because physiologic bone remodeling may compromise future implant placement by reducing the height and width of the alveolar ridge [8]. Socket preservation procedures utilizing bone grafting materials are commonly performed to minimize post-extraction ridge resorption and maintain adequate bone volume for subsequent implant-supported rehabilitation [9,10]. Recent systematic reviews and meta-analyses have demonstrated favorable clinical outcomes with xenogeneic bone graft materials, supporting their use for ridge preservation and bone regeneration before implant placement [11].

Dental implants have become a predictable treatment option for replacing missing teeth and restoring function through a staged treatment approach that includes site development, implant placement, osseointegration, and definitive prosthetic rehabilitation [12]. Implant-supported single crowns have demonstrated predictable long-term clinical outcomes when appropriate surgical, prosthetic, and loading protocols are followed [13]. Alveolar ridge preservation has also been recognized as an important component of comprehensive implant treatment planning because the maintenance of ridge volume may facilitate ideal implant positioning and restorative outcomes [14]. The relationship between the endodontic condition of adjacent teeth and implant success has also been investigated, emphasizing the importance of careful diagnosis and treatment planning before extraction and implant rehabilitation [15].

Although implant therapy has demonstrated high long-term survival rates, biological and technical complications may occur, highlighting the importance of appropriate case selection, meticulous surgical technique, and long-term maintenance [16]. Screw-retained implant-supported restorations have demonstrated favorable restorative outcomes and facilitate retrievability when maintenance or repair is required [17]. The use of scan bodies enables accurate transfer of implant position information to the laboratory and facilitates the fabrication of implant-supported restorations through a digital workflow [18]. Delayed implant placement following socket preservation has also demonstrated predictable long-term survival in appropriately selected patients [19]. Furthermore, advances in intraoral scanning technologies have improved the accuracy of implant position transfer, allowing for the precise fabrication of implant-supported restorations [20].

Although the principles of socket preservation and delayed implant placement are well established, relatively few case reports provide sequential radiographic documentation of the entire rehabilitation process following endodontic treatment failure. This case report highlights the educational value of staged implant rehabilitation by illustrating each phase of treatment, from extraction and ridge preservation to implant placement, digital impression acquisition, and delivery of the definitive restoration. The report emphasizes the role of socket preservation in maintaining adequate bone volume and facilitating predictable implant-supported rehabilitation following the extraction of a tooth with an unfavorable prognosis.

## Case presentation

A 46-year-old male patient classified as American Society of Anesthesiologists (ASA) physical status class I presented for assessment of a previously root canal-treated maxillary left first molar (tooth #14) due to persistent periapical pathology associated with the tooth. The patient’s medical history was unremarkable, and he reported no known drug allergies. Before the surgical procedure, the patient was prescribed amoxicillin 500 mg for a duration of seven days. The patient expressed concerns about the long-term prognosis and predictability of the tooth and requested a definitive treatment approach.

Clinical findings and diagnostic assessment

Clinical and radiographic examination demonstrated persistent periapical radiolucency involving the root apices, findings consistent with endodontic treatment failure and an unfavorable long-term prognosis (Figure 1).

**Figure 1:**
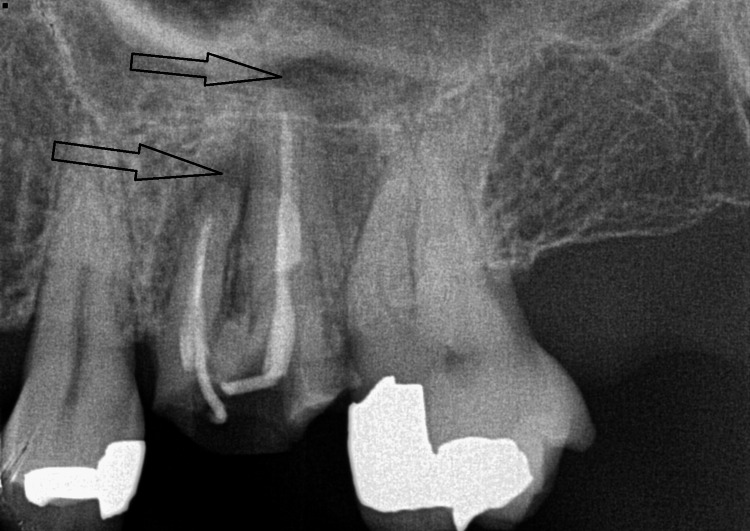
Initial periapical radiograph of tooth #14 demonstrating endodontic treatment failure The radiograph shows a previously endodontically treated maxillary left first molar (tooth #14). The arrows indicate a persistent periapical radiolucency associated with the root apices, findings consistent with endodontic treatment failure and an unfavorable long-term prognosis

The previous endodontic treatment had failed to resolve the periapical pathology, resulting in a poor long-term prognosis for the affected tooth. Treatment alternatives, including nonsurgical retreatment, endodontic microsurgery, and extraction followed by implant-supported rehabilitation, were discussed. After a comprehensive evaluation of the clinical and radiographic findings, together with consideration of the restorative prognosis, extraction of tooth #14 was selected as the preferred treatment approach.

Therapeutic intervention

The tooth was extracted atraumatically, and socket preservation was immediately performed using a bovine-derived bone graft material in combination with a collagen membrane to maintain alveolar ridge dimensions and facilitate future implant placement. Postoperative pain management included ibuprofen 600 mg every eight hours for three days. A postoperative periapical radiograph confirmed the appropriate placement of the graft material within the extraction socket (Figure 2).

**Figure 2:**
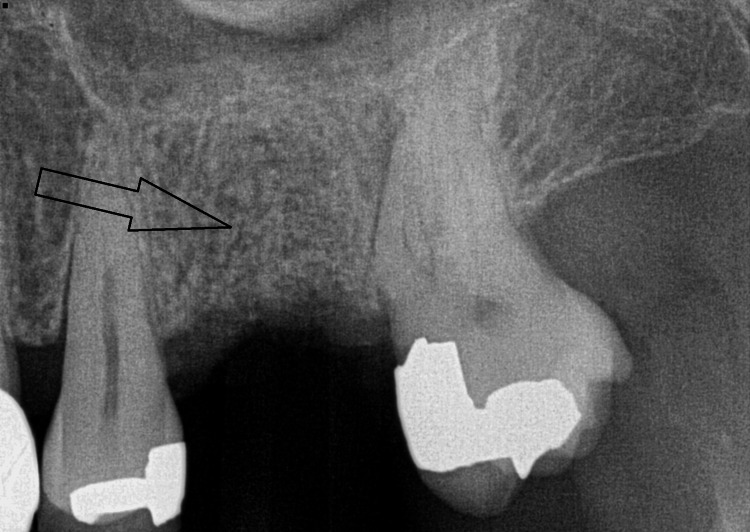
Post-extraction radiograph demonstrating socket preservation of tooth #14 Periapical radiograph obtained following extraction of the maxillary left first molar (tooth #14). The arrow indicates the radiopaque bone graft material placed within the extraction socket, consistent with alveolar ridge preservation performed to maintain ridge dimensions for future implant placement

The surgical site healed uneventfully without any postoperative complications, and a healing period of approximately three months was allowed to promote bone maturation within the preserved socket. Following the healing period, clinical and radiographic evaluations demonstrated satisfactory healing of the grafted site and adequate bone development for implant placement. A 5 × 9 mm dental implant was placed in the edentulous site according to the planned restorative position (Figure 3).

**Figure 3:**
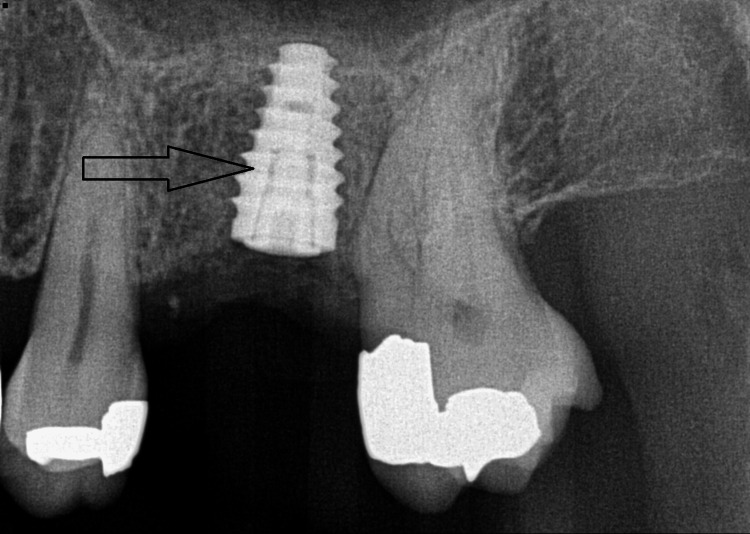
Periapical radiograph demonstrating implant placement at the site of tooth #14 Radiograph obtained following approximately three months of healing after extraction and socket preservation. The arrow indicates the dental implant placed in the edentulous site of the maxillary left first molar (tooth #14), demonstrating appropriate positioning within the healed alveolar ridge

An additional healing period of approximately three months was allowed to facilitate osseointegration. No intraoperative or postoperative complications were observed during this phase of treatment. After successful healing, a scan body was connected to the implant to obtain a digital impression for the fabrication of the definitive restoration (Figure 4).

**Figure 4:**
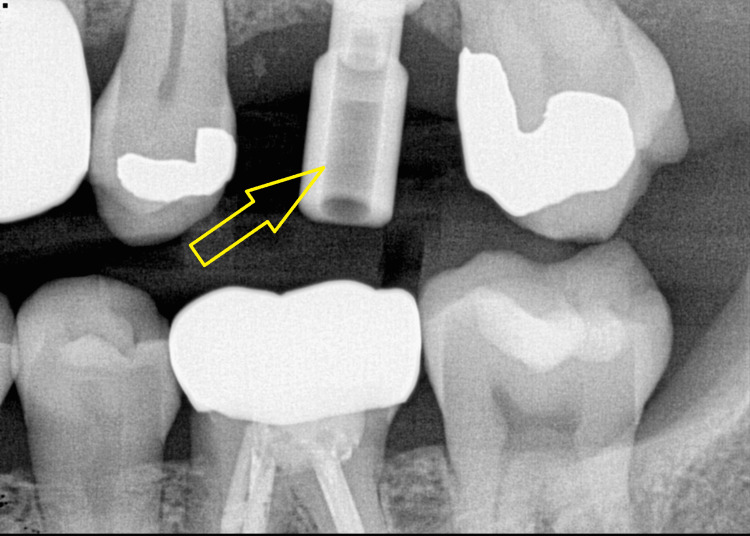
Scan body positioned on the implant at the site of tooth #14 Radiograph demonstrating a scan body connected to the dental implant during the digital impression phase. The arrow indicates the scan body, which was utilized to accurately transfer the implant position information for fabrication of the definitive implant-supported crown

The digital records were transferred to the dental laboratory for fabrication of the definitive implant-supported crown. Approximately three weeks later, the definitive implant-supported crown was delivered. The final periapical radiograph obtained at the time of restoration delivery demonstrated appropriate implant positioning, satisfactory adaptation of the definitive restoration, and successful rehabilitation of the edentulous site (Figure 5).

**Figure 5:**
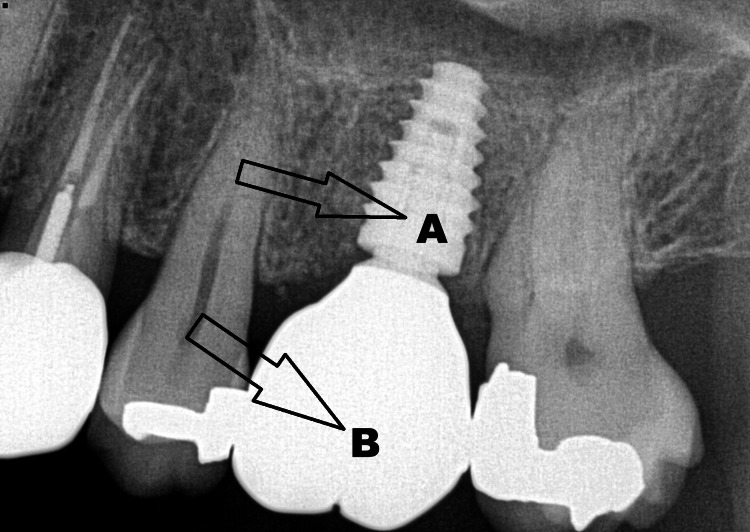
Final implant-supported crown restoration at the site of tooth #14 Periapical radiograph demonstrating the definitive implant-supported restoration following completion of the restorative phase. Arrow A indicates the dental implant, and Arrow B indicates the definitive implant-supported crown. Together, these findings demonstrate successful rehabilitation of the previously extracted maxillary left first molar (tooth #14) and completion of the staged implant rehabilitation

Follow-up and outcomes

The patient returned for a routine clinical follow-up three months after delivery of the definitive implant-supported crown. At that visit, the patient reported no complaints, and no biological or prosthetic complications were observed. Follow-up radiographs were not obtained. Serial radiographs documented the complete progression of treatment from endodontic failure through extraction, socket preservation, implant placement, digital impression acquisition using a scan body, and definitive implant-supported restoration. A timeline of clinical events is presented in Table 1.

**Table 1:** Timeline of clinical events

Time point	Clinical event
Initial presentation	Evaluation of a previously endodontically treated maxillary left first molar (tooth #14) with persistent periapical pathology and unfavorable prognosis
Preoperative period	Amoxicillin 500 mg prescribed for seven days
Day 0	Atraumatic extraction of tooth #14 and socket preservation using a bovine-derived bone graft and collagen membrane
Immediate postoperative period	Postoperative radiograph confirmed appropriate graft placement; ibuprofen 600 mg every eight hours prescribed for three days
3 months	Clinical and radiographic evaluation demonstrated satisfactory healing of the grafted site
3 months	Placement of a 5 × 9 mm dental implant
6 months	Completion of osseointegration period
6 months	Digital implant impression obtained using a scan body
6 months + 3 weeks	Delivery of the definitive implant-supported crown
9 months	Clinical follow-up visit with no biological or prosthetic complications reported

## Discussion

Endodontic treatment remains one of the most successful procedures for preserving natural dentition; however, treatment failure may occur despite appropriate therapy. Persistent intraradicular infection, inadequate canal disinfection, coronal leakage, missed canal anatomy, and persistent apical periodontitis are among the principal factors associated with endodontic failure [1-5]. When a previously treated tooth presents with persistent pathology or an unfavorable prognosis, extraction may become necessary [6,7]. In such situations, preservation of alveolar ridge dimensions becomes an important consideration because post-extraction bone resorption can complicate future implant placement and restorative outcomes [8].

Socket preservation procedures are commonly utilized to minimize ridge resorption and maintain adequate bone volume following tooth extraction [9,10]. Recent systematic reviews have also demonstrated favorable clinical outcomes with xenogeneic bone graft materials for preservation of extraction sockets and preparation of implant recipient sites [11]. In the present case, bone grafting was performed after extraction of the failed maxillary left first molar to facilitate future implant rehabilitation. Serial radiographs demonstrated healing of the grafted site and the development of sufficient bone volume for delayed implant placement [12].

In the present case, delayed implant placement was selected to allow healing of the grafted extraction site before rehabilitation of the edentulous space. Following a healing period of approximately three months, radiographic evaluation demonstrated satisfactory site development for implant placement. Implant-supported single crowns have demonstrated predictable clinical outcomes when appropriate surgical, prosthetic, and loading protocols are followed [13]. Although alternative treatment options, such as nonsurgical retreatment, endodontic microsurgery, intentional replantation, or continued clinical and radiographic monitoring, may be considered in carefully selected cases, extraction followed by staged implant rehabilitation was considered the most predictable treatment approach because of the persistent periapical pathology and the unfavorable long-term prognosis of the tooth. The sequential radiographs documented the transition from socket preservation to implant placement as part of the staged rehabilitation process.

Digital implant workflows have become increasingly common in restorative dentistry. Alveolar ridge preservation has been recognized as an important component of comprehensive implant treatment planning because maintaining ridge volume may facilitate ideal implant positioning and restorative outcomes [14]. The relationship between the endodontic condition of adjacent teeth and implant success has also been investigated, emphasizing the importance of careful diagnosis and treatment planning before extraction and implant rehabilitation [15]. Although dental implants demonstrate high long-term survival rates, clinicians should remain aware of potential biological and technical complications that may influence treatment outcomes [16]. Screw-retained implant-supported restorations have demonstrated predictable restorative outcomes and facilitate retrievability when maintenance or repair is required [17].

The use of scan bodies enables accurate transfer of implant position information to the laboratory and facilitates the fabrication of implant-supported restorations through a digital workflow [18]. Delayed implant placement has demonstrated favorable long-term survival when adequate healing of the extraction site is achieved before implant placement [19]. Furthermore, advances in intraoral digital implant scanning technologies have improved the accuracy of implant position transfer and prosthetic fabrication [20]. In the present case, careful treatment planning and staged rehabilitation resulted in uneventful healing without surgical or restorative complications during the documented treatment period. A clinical follow-up performed three months after delivery of the definitive implant-supported crown demonstrated satisfactory function of the restoration without biological or prosthetic complications; however, no follow-up radiographs were obtained.

The present report is limited by its nature as a single-patient case report, the relatively short clinical follow-up period after delivery of the definitive implant-supported crown, and the absence of follow-up radiographic evaluation. Nevertheless, the sequential radiographs provide a clear demonstration of the treatment stages involved in the rehabilitation of a failed endodontically treated maxillary first molar through extraction, socket preservation, implant placement, digital impression acquisition, and definitive implant-supported restoration. Although larger clinical studies with longer follow-up are necessary to further evaluate long-term outcomes, this case report illustrates the successful use of a staged treatment approach for rehabilitation following endodontic failure and highlights the value of sequential radiographic documentation in illustrating each stage of implant rehabilitation.

## Conclusions

This report demonstrates the radiographic progression of treatment for a failed endodontically treated maxillary left first molar managed through extraction, socket preservation, delayed implant placement, and definitive implant-supported rehabilitation. Sequential radiographs documented healing of the grafted site, successful implant placement after an appropriate healing period, digital impression acquisition using a scan body, and delivery of the definitive restoration. Although limited by the nature of a single-patient case report, the relatively short follow-up period, and the absence of follow-up radiographic evaluation, this report illustrates the successful application of a staged treatment approach following endodontic treatment failure. The principal clinical lesson is that preservation of alveolar ridge dimensions at the time of extraction may facilitate predictable implant placement and favorable restorative outcomes when implant rehabilitation is planned.
